# Overexpression of miR-199b-5p in Colony Forming Unit-Hill’s Colonies Positively Mediates the Inflammatory Response in Subclinical Cardiovascular Disease Model: Metformin Therapy Attenuates Its Expression

**DOI:** 10.3390/ijms25158087

**Published:** 2024-07-25

**Authors:** Sherin Bakhashab, Rosie Barber, Josie O’Neill, Catherine Arden, Jolanta U. Weaver

**Affiliations:** 1 Biochemistry Department, King Abdulaziz University, P.O. Box 80218, Jeddah 21589, Saudi Arabia; sbakhashab@kau.edu.sa; 2 Translational and Clinical Research Institute, Newcastle University, Newcastle upon Tyne NE2 4HH, UKjosieoneill@virginmedia.com (J.O.); 3 Center of Excellence in Genomic Medicine Research, King Abdulaziz University, P.O. Box 80216, Jeddah 21589, Saudi Arabia; 4 Biosciences Institute, Newcastle University, Newcastle upon Tyne NE2 4HH, UK; catherine.arden@newcastle.ac.uk; 5 Department of Diabetes, Queen Elizabeth Hospital, Gateshead, Newcastle upon Tyne NE9 6SH, UK; 6 Vascular Biology and Medicine Theme, Newcastle University, Newcastle upon Tyne NE1 7RU, UK

**Keywords:** miR-199b-5p, CFU-Hill colonies, subclinical CVD, metformin

## Abstract

Well-controlled type 1 diabetes (T1DM) is characterized by inflammation and endothelial dysfunction, thus constituting a suitable model of subclinical cardiovascular disease (CVD). miR-199b-5p overexpression in murine CVD has shown proatherosclerotic effects. We hypothesized that miR-199b-5p would be overexpressed in subclinical CVD yet downregulated following metformin therapy. Inflammatory and vascular markers were measured in 29 individuals with T1DM and 20 matched healthy controls (HCs). miR-199b-5p expression in CFU-Hill’s colonies was analyzed from each study group, and correlations with inflammatory/vascular health indices were evaluated. Significant upregulation of miR-199b-5p was observed in T1DM, which was significantly downregulated by metformin. miR-199b-5p correlated positively with vascular endothelial growth factor-D and c-reactive protein (CRP: nonsignificant). ROC analysis determined miR-199b-5p to define subclinical CVD by discriminating between HCs and T1DM individuals. ROC analyses of HbA1c and CRP showed that the upregulation of miR-199b-5p in T1DM individuals defined subclinical CVD at HbA1c > 44.25 mmol and CRP > 4.35 × 10^6^ pg/mL. Ingenuity pathway analysis predicted miR-199b-5p to inhibit the target genes *SIRT1*, *ETS1*, and *JAG1*. Metformin was predicted to downregulate miR-199b-5p via NFATC2 and STAT3 and reverse its downstream effects. This study validated the antiangiogenic properties of miR-199b-5p and substantiated miR-199b-5p overexpression as a biomarker of subclinical CVD. The downregulation of miR-199b-5p by metformin confirmed its cardio-protective effect.

## 1. Introduction

Atherogenesis has complex pathogenesis, although several common clinical occurrences between cardiovascular disease (CVD) and type 1 diabetes (T1DM) have been identified [1,2,3]. Early studies of T1DM pathogenesis in nonobese diabetic mouse models, and more recently in human trials, have attributed the pathophysiological conjunction to hyperglycemia, dyslipidemia, increased inflammation, and endothelial dysfunction [4,5,6,7,8]. These pathways have been associated with the accelerated development of atherosclerosis and increased risk of CVD in T1DM individuals [6,9]. Moreover, recent data estimated a standard CVD-related mortality ratio of 5.7 and 11.3 for men and women with T1DM, respectively [10], and revealed a 10-fold elevated risk of CVD-related mortality in T1DM individuals overall [11]. It is evident that T1DM individuals with poor glycemic control immediately present an elevated risk of developing CVD; nonetheless, cardio-detrimental changes at the cellular level are becoming increasingly apparent, even in those with good glycemic control. Collectively, this research proposes that well-controlled T1DM can be utilized as a model of subclinical CVD.

Inflammation is the most prominent pathological event leading to atherosclerosis development in T1DM individuals [12], as chronic inflammation of the vascular wall ultimately leads to acute thrombosis and fibrous cap rupture [8,9]. C-reactive protein (CRP) is a well-established high-sensitivity marker of inflammation and is significantly upregulated in T1DM individuals [13]. Multiple studies have linked CRP with critical pathways in CVD development and demonstrated the direct influences it has on proatherogenic mechanisms, including the activation of cytokine release from endothelial cells (ECs), vascular smooth muscle cells, and macrophages, as well as the increased uptake of low-density lipoprotein and foam cell formation and the amplification of innate immunity [14,15,16].

Previous studies have suggested that CVD risk factors contribute to vascular injury and induce endothelial dysfunction, which is believed to initiate the pathological mechanisms involved in the progression of atherosclerosis. As first described by Asahara et al. [17], the migration of circulating endothelial progenitor cells (cEPCs) from the vascular intima following arterial damage promotes inflammation and arterial stenosis [1,18]. cEPCs are characterized by the expression of hematopoietic stem cell markers CD34+, CD133+, and/or vascular endothelial growth factor receptor-2^+^ (VEGFR-2^+^), which are critical to vasculogenesis and integrity [2,19,20]. By consequence, studies correlating reduced cEPC activity and abundancy with increased CVD risk have validated their use as CVD biomarkers [1,20,21]; most notably, Hill et al. [2] found a significant inverse correlation between cEPC levels and the Framingham Risk Score (total burden of coronary heart disease risk factors at 10 years).

Interestingly, high circulating ECs (cECs) and low cEPC levels have also been associated with T1DM, thus demonstrating direct and inverse correlations with HbA1c levels, respectively [13,19,22]. Sibal et al. [13] reported that the relationship between low cEPC levels and elevated inflammatory markers in T1DM develops prior to clinical presentation of vascular damage, thus proposing endothelial dysfunction as an initiator of atherosclerosis development and supporting the use of cEPCs as CVD biomarkers.

The colony forming unit (CFU)-Hill’s colony assay developed by Hill et al. [2] facilitates the isolation of two distinct cEPC populations from peripheral blood mononuclear cells (PBMNCs): (i) Proangiogenic cells (PACs) are cultured from adherent PBMNCs and secrete angiogenic cytokines, while (ii) CFU-Hill’s colonies are derived from replated nonadherent PBMNCs and exhibit high EPC proliferation potency [2,23,24]. The contributions of these characteristics to vasculogenesis/angiogenesis emphasize the role of endothelial function in vascular health, though they have also been implicated in diabetes following studies that indicated reduced levels of both PACs and CFU-Hill’s colonies in individuals with T1DM against controls [1]. Furthermore, Loomans et al. [19] reported impaired PAC function in individuals with T1DM, as well as an inverse correlation between PAC count and HbA1c levels, and a significant reduction in CFU-Hill’s colonies was observed by Hill et al. [2] in patients with hyperlipidemia, hypercholesterolemia, hypertension, and/or diabetes.

There is accumulating evidence validating the involvement of micro-RNAs (miRNAs) in regulating CVD pathogenicity. miRNAs are highly conserved endogenous noncoding RNAs (21–24 nucleotides) transcribed as precursors by RNA polymerase II that regulate gene expression post-transcriptionally [5,25]. Derived from the miR-199 family, miR-199b-5p is one of many miRNAs explored to potentially possess regulatory control within CVD development [26,27]. miR-199b-5p was shown to be associated with T2DM [28] and highly responsive to glucose stimulation in murine beta cells [29]. Studies have demonstrated the upregulation of miR-199b-5p expression in mouse models of heart failure, in addition to inhibition of endothelial proliferation and vascular formation in mouse cardiac microvascular cells [26,30]. Together, these data suggest an antiangiogenic role of miR-199b-5p, thus making it a promising miRNA to explore further in this study.

Metformin is an antidiabetic biguanide drug that benefits glucose metabolism and improves glycemic control in T2DM patients by reducing hepatic gluconeogenesis [31,32]. The cardio-protective benefits of metformin therapy are well documented to reduce cardiovascular-related mortality and the incidence of cardiovascular events. Recently, the REMOVAL Trial identified reduced atherosclerotic progression in individuals with T1DM undergoing metformin treatment, thus suggesting direct antiatherogenic effects and promoting metformin use in CVD risk management in T1DM [33]. The MERIT Trial showed the cardio-protective effect of metformin through improving cEPCs, CFU-Hill’s colonies, cECs, and PACs counts and functions in T1DM individuals [1]. Furthermore, multiple studies have predicted a cardio-protective role of metformin via miRNA regulation [34,35]. Liang et al. reported that metformin increased atherosclerotic plaque stability in T1DM *ApoE^−/−^* mice by regulating miR-124 expression, thus leading to prevent CVD progression [36]. Although explored by many studies, the growing prevalence of CVD signifies the importance of identifying novel biomarkers to facilitate the early identification and management of atherosclerotic progression. Meanwhile previous research has highlighted the antiangiogenic effect of miR-199b-5p, thus establishing its role in emerging T1DM-related CVD, which is paramount to understanding its potential as a therapeutic target for metformin. Therefore, we aimed in this study to investigate the role of miR-199b-5p in subclinical CVD and explored its therapeutic relationship with metformin.

## 2. Results

### 2.1. Clinical and Metabolic Characteristics of Study Participants

The substudy undertaken for this project enrolled 29 individuals with T1DM as the treatment group (TG) and 20 age- and sex-matched healthy controls (HCs), with a mean age of 47 ± 13 and 46 ± 12 years, respectively. The study was conducted in males and females (9 males and 11 females in HCs; 14 males and 15 females in T1DM). The T1DM individuals had a mean diabetes duration of 22 ± 14 years, relatively stable glycemic control (HbA1c: 57.3 ± 7.6 mmol/mol), and no previous history of CVD. The baseline demographic, as well as the clinical and metabolic characteristics of the TG and HCs, were previously published by us [37].

### 2.2. Comparisons of Inflammatory Markers and Vascular Health Parameters between T1DM Individuals and HCs

The data on inflammatory and vascular health markers in this cohort had been previously published [1,35,38,39,40]. Briefly, the plasma levels of CRP (6.78 × 10^6^ pg/mL; *p* < 0.001), VEGF-D (206.2 ± 71.18 pg/mL, *p* = 0.002), tumor necrosis factor (TNF)-α (0.3899 pg/mL; *p* = 0.041), and interleukin (IL)-10 (0.1528 pg/mL; *p* = 0.008) were significantly higher in T1DM individuals compared to HCs. Furthermore, a significant upregulation of soluble thrombomodulin (842.2 ± 379.4 ng/mL; *p* = 0.04) and receptor tyrosine kinase (Tie2; 744.6 ± 280.3 ng/mL; *p* = 0.014) expression was observed in comparison between HCs and T1DM individuals. Significant decreases in VEGF levels (−10.61 ± 4.44 pg/mL; *p* = 0.025) were observed in T1DM individuals versus HCs. The serum levels of insulin-like growth factor-1 (IGF-1) were significantly lower in T1DM individuals compared to HCs (−0.050 ng/mL; *p* = 0.0015), whereas there was no significant difference in IGF-binding protein-3 (IGFBP-3) levels between T1DM individuals and HCs, as previously shown in [38].

The vascular health parameters were significantly lower in T1DM individuals than in HCs, including cEPCs, i.e., CD34^+^ (*p* < 0.0001) and CD34^+^CD133^+^ (*p* = 0.013) per 100 lymphocytes, as well as CFU-Hill’s colonies (*p* = 0.04) and PACs (*p* < 0.001).

### 2.3. Expression of miR-199b-5p between Study Groups

**Figure 1:**
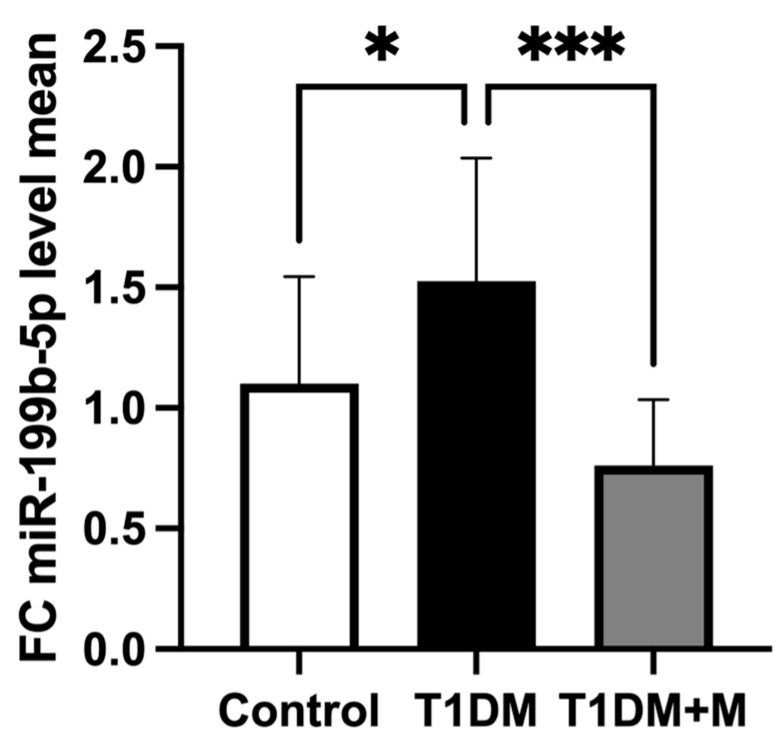
The comparison of miR-199b-5p expression in CFU-Hill’s colonies between healthy controls and T1DM individuals before (T1DM) and after (T1DM + M) metformin treatment. Fold change between miR-199b-5p mean 2^(ΔΔCq)^ values used as a measure of expression. Data were analyzed by one-way ANOVA followed by Tukey test and presented as means ± SD: * *p* < 0.05, *** *p* < 0.001.

The expression of miR-199b-5p in CFU-Hill’s colonies was found to be significantly upregulated by a 1.5-fold change (*p* = 0.04) in T1DM individuals compared to HCs (Figure 1). Following eight weeks of metformin therapy, significantly lower miR-199b-5p expression levels (−1.4-fold change; *p* < 0.001) were observed in T1DM individuals, thereby normalizing them in comparison to HCs (*p* > 0.05; nonsignificant).

### 2.4. Correlations Trends of miR-199b-5p with Inflammatory Markers and Vascular Health Parameters

Linear regression analyses of inflammatory markers and vascular health parameters identified that miR-199b-5p expression in CFU-Hill’s colonies demonstrated a trend towards a positive correlation with the plasma levels of thrombomodulin (r^2^ = 0.1226; *p* = 0.0934) and TNF-α (r^2^ = 0.1264; *p* = 0.0959). Trends towards a negative correlation were observed between miR-199b-5p and CD34^+^ (r^2^ = 0.0070; *p* = 0.6967), and between miR-199b-5p and CFU-Hill’s colonies (r^2^ = 0.0797; *p* = 0.1813). These data were all found to be nonsignificant, possibly due to the small sample sizes. The levels of VEGF, IGF-1, and IGFBP-3 were not significantly correlated with the miR-199b-5p expression levels in CFU-Hill colonies (r^2^ = 0.0342 and *p* = 0.387; r^2^ = 0.0307 and *p* = 0.4239; r^2^ = 0.0036 and *p* = 0.7867, respectively).

### 2.5. Analysis of miR-199b-5p, Inflammatory Markers, and Vascular Health Parameters as Diagnostic Markers

**Figure 2:**
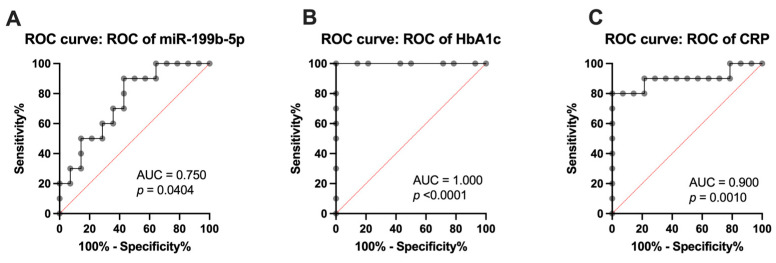
Receiver operating characteristic (ROC) curve analysis of (**A**) miR-199b-5p in discriminating between healthy controls and T1DM individuals (AUC = 0.750; *p* = 0.0404), (**B**) HbA1c (AUC= 1.000; *p* < 0.0001), and (**C**) CRP (AUC = 0.900; *p* = 0.0010). ROC curve analysis was performed to determine optimal cutoff values. HbA1c: glycated hemoglobin; CRP: c-reactive protein.

Receiver operating characteristic (ROC) curve analysis of the miR-199b-5p in CFU-Hill’s colonies demonstrated its ability to distinguish between healthy and T1DM subjects, thus signifying its applicability as a biomarker for subclinical CVD. The area under the curve (AUC) value (0.7500; *p* = 0.0404) indicated miR-199b-5p as a good model for discriminating between HCs and T1DM individuals, thereby yielding a cutoff value of −4.234-fold (sensitivity = 90%; specificity = 57.14%), as shown in Figure 2A.

ROC curve analyses of HbA1c (Figure 2B) and CRP (Figure 2C) were performed to determine the levels at which miR-199b-5p was upregulated in T1DM individuals. It was found that the significant upregulation of miR-199b-5p (AUC= 1.000; *p* < 0.0001) defined subclinical CVD at HbA1c > 44.25 mmol/mol (sensitivity = 100%; specificity = 100%). Comparably, the significant upregulation of miR-199b-5p (AUC = 0.9000; *p* = 0.0010) defined subclinical CVD at CRP > 4.35 × 10^6^ pg/mL (sensitivity = 80%; specificity = 100%). Moreover, linear regression analysis revealed tendencies (but not significant) towards a positive correlation of miR-199b-5p with both HbA1c (r^2^ = 0.0663; *p* = 0.2245) and CRP (r^2^ = 0.1483; *p* = 0.0632) before metformin intervention.

**Figure 3:**
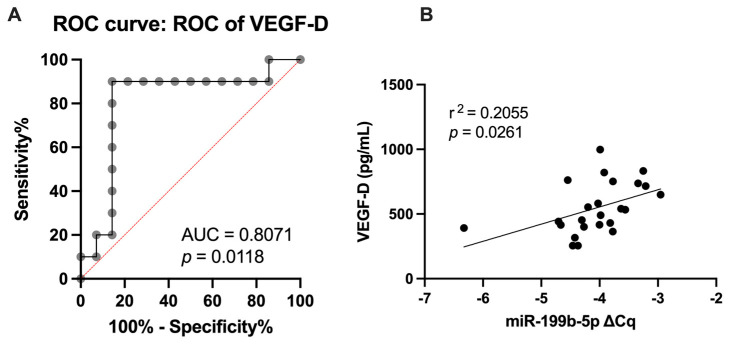
The relationship between miR-199b-5p expression in CFU-Hill’s colonies and plasma levels of VEGF-D. (**A**) Receiver operating characteristic (ROC) curve analysis of VEGF-D. (**B**) Correlation between miR-199b-5p expression in CFU-Hill’s colonies and plasma levels of VEGF-D. ROC curve analysis was performed to determine optimal cutoff values, while correlations were assessed using linear regression analysis. VEGF: vascular endothelial growth factor.

Supplementary ROC curve analyses of inflammatory markers and vascular health parameters revealed VEGF-D (Figure 3A) as an indicator of subclinical CVD, thus exhibiting significant upregulation of miR-199b-5p (AUC = 0.8071; *p* = 0.0118) and defining subclinical CVD at VEGF-D > 536.9 pg/mL (sensitivity = 90%; specificity = 85.71%). In addition, a significant positive correlation was observed between miR-199b-5p expression and VEGF-D plasma levels (Figure 3B) following linear regression analysis (r^2^ = 0.2055; *p* = 0.0261).

### 2.6. Predicted Molecular Targets and Functional Pathways of miR-199b-5p

**Figure 4:**
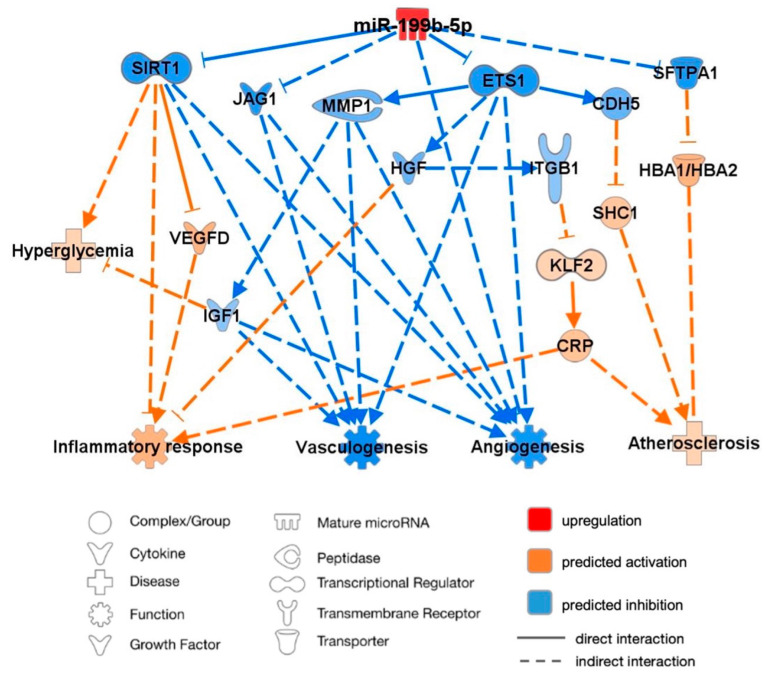
Ingenuity Pathway Analysis (IPA) prediction network of miR-199b-5p using this study’s data and its mRNA targets supporting its association with cardiovascular disease. Four pathways were considered relevant in this study: inflammatory response, vasculogenesis, angiogenesis, and atherosclerosis. Red signifies upregulation of miR-199b-5p, orange signifies predicted activation, and blue signifies predicted inhibition. Solid lines represent a direct interaction, and dashed lines represent an indirect interaction. All interactions have a value of *p* < 0.05. CDH5: cadherin 5; CRP: c-reactive protein; ETS1: ETS proto-oncogene 1, transcription factor; HBA1/HBA2: glycated hemoglobin subunit α1/2; HGF: hepatocyte growth factor; IGF1: insulin-like growth factor 1; ITGβ1: integrin subunit β1; JAG1: jagged canonical notch ligand 1; KLF2: Krüppel-like factor 2; MMP1: matrix metallopeptidase 1; SFTPA1: surfactant protein A1; SHC1: SHC-transforming protein 1; SIRT1: sirtuin 1; VEGFD: vascular endothelial growth factor D.

An analysis of predicted miR-199b-5p targets and pathways was performed using the knowledge-based database IPA to better understand the interactions of miR-199b-5p supporting its association with CVD, as illustrated in Figure 4. The mature miR-199a-5p shares the seed CCAGUGU with miR-199b-5p and thus was inputted into the IPA software alongside CRP, HbA1c, VEGF-D, and hyperglycemia to imitate a diabetic state. miR-199b-5p was subsequently predicted to have proatherosclerotic and hyperglycemic effects and enhance the inflammatory response while demonstrating a predicted inhibition of angiogenesis and vasculogenesis.

Through published knockout and correlation studies, the overexpression of miR-199b-5p was proven to directly inhibit the mRNA expression of sirtuin-1 (SIRT1) and ETS proto-oncogene 1 (ETS1), thus resulting in the inhibition of angiogenesis and vasculogenesis. The inhibition of SIRT1 was predicted to activate VEGFD, both of which led to the activation of the inflammatory response. ETS1 inhibition was predicted to inhibit hepatocyte growth factor (HGF), thus further activating inflammation and leading to the activation of Krüppel-like factor 2 (KLF2) through the inhibition of integrin-b1 (ITGb1). KLF2 activation was predicted to activate CRP, which was subsequently associated with the enhanced progression of atherosclerosis and inflammation. Cadherin-5 (CAD5) was also predicted to be inhibited by ETS1, thus leading to the predicted activation of proatherosclerotic SHC-transforming protein-1 (SHC1). Additionally, the predicted inhibition of matrix metalloprotein-1 (MMP*1*) by ETS1 inhibited IGF1, thus resulting in the predicted inhibition of angiogenesis and vasculogenesis. Inhibited IGF1 was also predicted to enhance hyperglycemia. The indirect inhibition by upregulated miR-199b-5p of jagged-1 (JAG1) was also demonstrated, thus similarly resulting in the inhibition of angiogenesis and vasculogenesis. Finally, miR-199b-5p upregulation was predicted to lead to the activation of HbA1c (HBA1/HBA2) through the inhibition of surfactant protein A1 (SFTPA1), thus resulting in a predicted association with enhanced atherosclerosis progression.

### 2.7. miR-199b-5p Predicted Targets and Pathways Following Metformin Intervention

**Figure 5:**
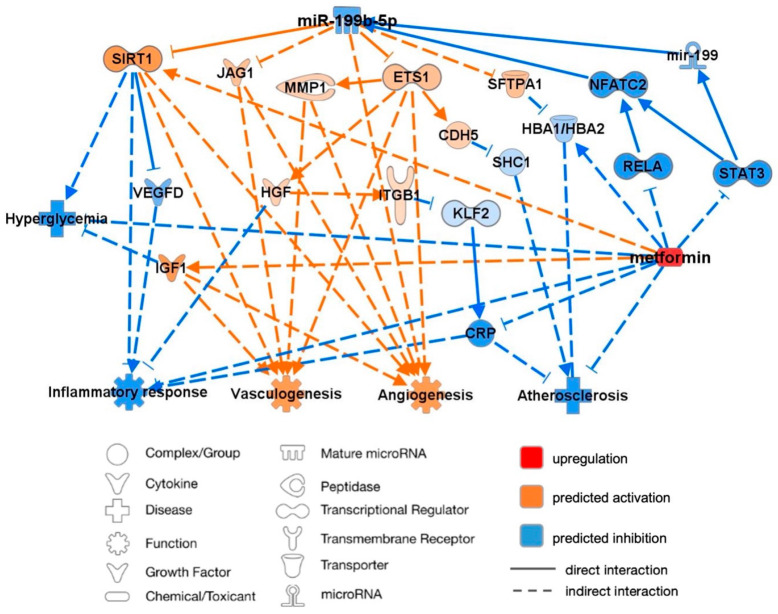
Ingenuity Pathway Analysis (IPA) prediction network of miR-199b-5p molecular targets and functional pathways following metformin intervention. Red signifies upregulation of metformin, orange signifies predicted activation, and blue signifies predicted inhibition. Solid lines represent a direct interaction, and dashed lines represent an indirect interaction. All interactions have a value of *p* < 0.05. CDH5: cadherin 5; CRP: c-reactive protein; ETS1: ETS proto-oncogene 1, transcription factor; HBA1/HBA2: glycated hemoglobin subunit α1/2; HGF: hepatocyte growth factor; IGF1: insulin-like growth factor 1; ITGβ1: integrin subunit β1; JAG1: jagged canonical notch ligand 1; KLF2: Krüppel-like factor 2; MAPK8: mitogen-activated protein kinase 8; mir-199: micro-RNA-199; MMP1: matrix metallopeptidase-1; NFATC2: nuclear factor of activated T cells 2; RELA: RELA proto-oncogene, NF-κB subunit; SFTPA1: surfactant protein A1; SHC1: SHC-transforming protein 1; SIRT1: sirtuin 1; STAT3: signal transducer and activator of transcription 3; VEGFD: vascular endothelial growth factor D.

The treatment of T1DM individuals with metformin resulted in the downregulation of miR-199b-5p expression through the regulation of various transcriptional regulators. In opposition to before, this led to the activation of SIRT1, JAG1, SFTPA1, and ETS1. The predicted inhibition of the signal transducer and activator of transcription-3 (STAT3) was predicted to inhibit the mir-199 and nuclear factor of activated T cells 2 (NFATC*2*), which in turn would directly inhibit miR-199b-5p. NFATC2 was also inhibited through the predicted inhibition of RELA proto-oncogene. As illustrated in Figure 5, functional pathway analysis predicted metformin intervention to ultimately enhance vasculogenesis and angiogenesis while reducing the progression of hyperglycemia, atherosclerosis, and the inflammatory response.

## 3. Discussion

**Figure 6:**
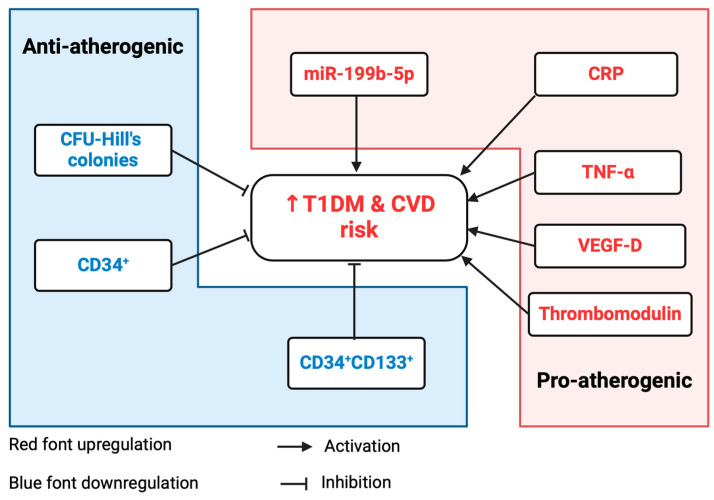
Schematic summary of the findings of this research following the study in T1DM individuals with subclinical CVD. Upregulated miR-199b-5p, CRP, TNF-α, and thrombomodulin demonstrated proatherogenic effects and contributed to increased CVD risk in T1DM individuals. Downregulation of antiatherogenic CFU-Hill’s colonies, CD34^+^ stem cells, and CD34^+^CD133^+^ stem cells further contributed to elevated T1DM-related CVD risk. Upregulation of VEGF-D proposed a pro-atherosclerotic role in T1DM. Created using BioRender.com. T1DM: type 1 diabetes mellitus; CVD: cardiovascular disease; miR: micro-RNA; CRP: c-reactive protein; TNF: tumor necrosis factor; CD: cluster of differentiation; VEGF: vascular endothelial growth factor. The red color denoted increased and blue color decreased.

The upregulation of miR-199b-5p supported its antiangiogenic effects previously reported in animal studies, and its negative association with vascular health parameters highlighted its role in the pathogenicity of CVD. A summary of these findings is depicted in Figure 6.

**Figure 7:**
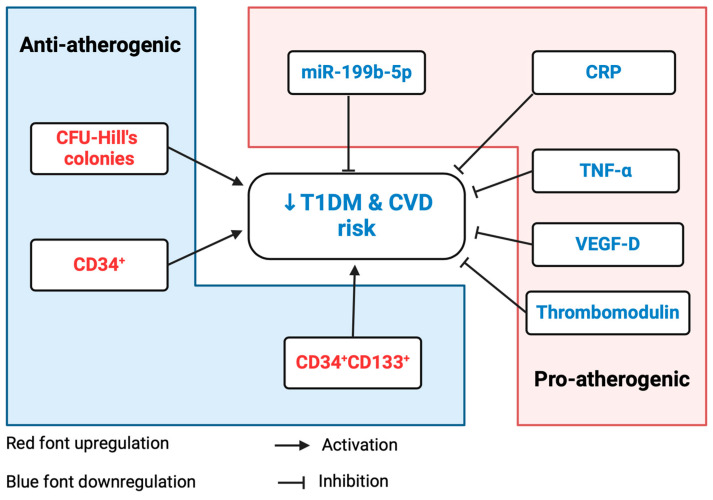
Schematic summary of the findings of this research following metformin intervention. Metformin inhibited miR-199b-5p and subsequently downregulated CRP and TNF-α, thus contributing to reduced CVD risk in T1DM individuals. Upregulation of antiatherogenic CFU-Hill’s colonies, CD34^+^ stem cells, and CD34^+^CD133^+^ stem cells further contributed to reduced T1DM-related CVD risk. Downregulated thrombomodulin reflected the reduced level of inflammation. Downregulation of VEGF-D reduced its proatherosclerotic role in T1DM. Created using BioRender.com. T1DM: type 1 diabetes mellitus; CVD: cardiovascular disease; miR: micro-RNA; CRP: c-reactive protein; TNF: tumor necrosis factor; CD: cluster of differentiation; VEGF: vascular endothelial growth factor. The red color denoted increased and blue color decreased.

This study also demonstrated the cardio-protective role of metformin in a diabetic model, thus reporting the downregulation of miR-199b-5p via canonical pathways and therefore reversal of its detrimental effects; this is summarized in Figure 7. Metformin had been demonstrated as an atherogenic disruptor of atherosclerotic risk factors and for its role in protecting the arteries from fibrosis and remodeling [41]. There is accumulating evidence that metformin directly influences atherosclerosis by improving endothelial function independently of its hypoglycemic effect while simultaneously exhibiting proangiogenic behavior [1,42,43]. Moreover, we have shown the cardio-protective effect of metformin in patients with T1DM through the improvement in CFU-Hill colonies, cEPCs, cECs, and PACs without changes in the glycemic control [1,37,40].

The potential effect of metformin as an endocrine disruptor was detected on sex-specific reproductive changes in humans, rodents, and fish [44,45,46]. Recent research has found a link between the number of male genital birth abnormalities and the parental use of metformin. It has been shown that metformin decreases the amount of circulating testosterone in both men and women [46]. Metformin administration in utero in rats caused sex-specific reproductive alterations with decreased fertility, and metformin exposure in fish caused intersex results that were observed in testicular tissue in experimental animal models [45,46]. There has been no research performed on the effect of metformin as an endocrine disruptor at the cardiovascular level.

### 3.1. Upregulation of miR-199b-5p Expression in T1DM

This study is the first to investigate miR-199b-5p expression in CFU-Hill’s colonies, thus reporting significant upregulation in T1DM individuals. This is in concordance with previous studies that have associated an altered expression of miR-199b-5p with T2DM individuals, in addition to increased expression in pancreatic the islets removed from diabetic mice and in the plasma from diabetic patients [29].

Previous studies have demonstrated the upregulation of miR-199b-5p in animal models of heart failure and diabetic cardiomyopathy [26,30], as well as correlations with vascular damage in patients at high risk for developing CVD [47]. Specifically, the overexpression of miR-199b-5p in mouse and human heart failure has been shown to target calcineurin/NFATC gene expression. Mutant mice overexpressing miR-199b-5p presented insensitivity to calcineurin/NFATC signaling and reduced Dyrk1a expression, thus resulting in the development of stress-induced cardiomegaly. Furthermore, the in vivo inhibition of miR-199b-5p in mouse models of heart failure caused the distinct inhibition and reversal of hypertrophy and fibrosis [30].

Chan et al. reported miR-199b-5p overexpression to exhibit antiangiogenic characteristics through the negative regulation of *ETS1* and *MMP1* [48]. *ETS*^−/−^ mice displayed reduced wound blood flow and EC counts; thus, it was inferred that miR-199b-5p inhibits wound angiogenesis through the repression of the ETS1/MMP1 pathway [48]. This is consistent with IPA analysis in the present study, which predicted the direct inhibition of *ETS1* following miR-199b-5p overexpression, thus leading to hindered angiogenesis and vasculogenesis. This research validates these findings and suggests a pivotal role of miR-199b-5p in the pathogenic pathways leading to CVD, including disrupted vascular signaling, remodeling, and angiogenesis.

The ROC curve analysis indicated miR-199b-5p as a potential prognostic/diagnostic biomarker of T1DM/subclinical CVD, thus achieving an AUC of 0.7500 (90% sensitivity; 57.14% specificity [*p* = 0.0404]). Werneck-de-Castro et al. reported the high receptiveness of miR-199b-5p to glucose stimulation; hence, it was hypothesized that the difference in expression could be correlated with the presence of hyperglycemia [29]. To categorize the value at which miR-199b-5p upregulation occurred, ROC curve analysis was used to assess the cutoff value of HbA1c. The significant upregulation of miR-199b-5p was found to define subclinical CVD at an HbA1c > 44.25 mmol/mol (6.2%), thus indicating that increased CVD risk coincides with the prediabetes range (HbA1c 6.0–6.49%). These findings emphasize the validity in utilizing miR-199b-5p as a sensitive biomarker for subclinical CVD, even prior to clinical presentation.

### 3.2. Association between miR-199b-5p and HbA1c

Our linear regression analysis revealed that miR-199b-5p displayed a tendency towards a positive correlation with HbA1c, thus suggesting that the regulation of miR-199b-5p may be influenced by diabetic control. These data concur with those of Werneck-de-Castro et al., which conveyed that glucose actively upregulated miR-199 family expression in mouse pancreatic β-cells [29]. Moreover, the IPA analysis predicted that miR-199b-5p overexpression leads to indirect activation of hyperglycemia via *SIRT1* inhibition. In contrast, miR-199b-5p upregulation has been associated with enhanced β-cell proliferation, thus by consequence ameliorating diabetic pathogenicity [49]; thus, additional research is required to validate the relationship of miR-199b-5p with glucose metabolism.

### 3.3. Positive Association between miR-199b-5p and Inflammatory Markers

The findings of this study correspond to the correlation of T1DM individuals with inflammatory markers reported by previous studies [35,39,40]. Here, significantly increased levels of CRP, VEGF-D, and TNF-α were identified in T1DM individuals in comparison to the HCs. We also measured the soluble thrombomodulin, which had been detected as a potential marker of endothelial injury and risk factor of coronary heart disease [50,51].

#### 3.3.1. CRP

Intriguingly, this study is the first to report a trend towards a positive correlation between miR-199b-5p and CRP. The positive correlation indicated at present was validated by the IPA analysis of miR-199b-5p overexpression, thus predicting the upregulation of CRP via the direct inhibition of *ETS1*. CRP is widely established as a highly sensitive inflammatory marker; thus, its circumstance of upregulation in T1DM individuals confirms T1DM individuals being in an inflammatory state.

Considering this, the ROC curve analysis subsequently clarified CRP as an exceptionally sensitive biomarker of T1DM/subclinical CVD, thus yielding an AUC of 0.9000 (80% sensitivity; 100% specificity). Moreover, this indicates that the significant upregulation of miR-199b-5p defines subclinical CVD at CRP > 4.35 × 10^6^ pg/mL. The direct effect of CRP in atherosclerotic development has been recounted in several publications, thus highlighting that its function expands further than as merely a marker [14,15,16]. Specifically, Pasceri et al. ascertained that CRP ≥ 5 × 10^6^ pg/mL induced high expression levels of ICAM-1, VCAM-1, and E-selectin in coronary artery ECs, thus significantly enhancing inflammation [15]. There were several clinical trials exploring the effect of anti-inflammatory treatments on CVDs and CRP, including the JUPITER study [52] and CANTOS study [53] on patients who remained at increased vascular risk due to elevated levels of sCRP (>2 × 10^6^ pg/mL).

#### 3.3.2. VEGF-D

This study is the first to identify a significant direct correlation between miR-199b-5p and VEGF-D, which was subsequently supported by IPA analysis. miR-199b-5p overexpression was predicted to upregulate VEGF-D via the direct inhibition of SIRT1, thus advancing the inflammatory response. Considering its significant correlation with miR-199b-5p, the ROC curve analysis of VEGF-D achieved an AUC of 0.8071 (90% sensitivity; 85.71% specificity [*p* = 0.0118]), thus revealing its potential as a sensitive biomarker for T1DM/subclinical CVD. Studies have identified constitutive VEGF-D expression in both healthy and atherosclerotic arteries, most prominently in vascular smooth muscle cells, thus suggesting its role in vascular homeostasis [54]. Elevated VEGF-D levels in sera were detected to independently predict all-cause mortality in patients with suspected or known coronary artery disease [55]. In addition, increased VEGF-D was also associated with atrial fibrillation and ischemic stroke [56]. Diabetic rabbits showed the upregulation of VEGF-D and increased macrophages in atherosclerotic lesions, thus implicating its contribution to plaque neovascularization [54,57].

### 3.4. Downregulation of miR-199b-5p Expression Following Metformin Intervention

The effect of metformin on miR-199b-5p expression in CFU-Hill’s colonies was explored for the first time in this study. Currently, metformin is widely utilized as the first-line medical treatment for T2DM individuals, as it ameliorates hyperglycemia, but it is used less commonly in T1DM individuals due to the requirement for insulin supplementation. Studies have previously highlighted the cardio-protective role of metformin in diabetes [33,58,59,60], including its effects on pro-/antiatherogenic miRNAs.

This study validates preceding research that demonstrates the cardiovascular benefit of metformin therapy in T1DM, thus indicating that metformin significantly downregulates miR-199b-5p expression in T1DM individuals to the extent of normalization. This is concordant with a previous publication that reported the suppression of miR-199b-5p expression in human melanoma cells following metformin treatment [61]. Additionally, these findings strengthen evidence that supports metformin cardio protection via miRNA regulation. Studies exploring antiangiogenic miR-222, miR-195, and miR-21a [34], as well as proatherogenic miR-18a-5p [35], reported unanimous upregulation in T1DM individuals. Comparably, these miRNAs were defined as biomarkers of subclinical CVD and were significantly downregulated by metformin.

### 3.5. Prediction Model: Pathway Analysis of miR-199b-5p in Relation to Cardiovascular Function

The IPA prediction model of miR-199b-5p summarized the findings of this study and predicted that the overexpression of miR-199b-5p directly inhibits *SIRT1* and *ETS1* and indirectly inhibits *JAG1*. In accordance with the miRNA/mRNA correlation analyses, this was predicted to ultimately upregulate the levels of CRP, HbA1c, and VEGF-D. miR-199b-5p overexpression was therefore predicted to be cardio-detrimental through diminished angiogenesis and vasculogenesis, as well as accelerated inflammation and atherosclerosis. Additionally, the downregulation of *IGF1* via the ETS1/MMP1 pathway in this prediction model contributed to hyperglycemia, which has been validated by studies on the b-cells of diabetic transgenic mice [62]. The upregulation of *HBA1* was predicted through the downregulation of *SFTPA1*, which has previously been associated with reduced glycemic levels and anti-inflammatory effects [63].

#### 3.5.1. Angiogenesis and Vasculogenesis

The predicted inhibition of *SIRT1*, *ETS1*, and *JAG1* was associated with reduced angiogenesis and vasculogenesis, as confirmed in previous knockout studies. *SIRT1* is highly expressed in vascular ECs, where it uniquely regulates angiogenic signaling [64]; hence, studies on *SIRT1*^−/−^ mice have demonstrated defective blood vessel formation and reductions in circulating hematopoietic progenitors [64,65]. The upregulation of proangiogenic genes has been shown to be largely dependent on functional ETS1 [66], with *ETS*^−/−^ mice exhibiting reduced vascular ECs and stunted wound angiogenesis [48]. The predicted inhibition of *MMP1*, and subsequently *IGF1*, in relation to *ETS1* was similarly associated with antiangiogenic/antivasculogenic effects; this is consistent with the high MMP1 activity found in vascular remodeling [67] and corresponds to studies on murine models that identified IGF1 as a proangiogenic factor in myocardial microvascular ECs [68]. JAG1 is fundamental in vascular remodeling and regulates VSMC proliferation and differentiation by mediating notch signaling; thus, *JAG1*^−/−^ murine models developed cardiac anomalies and defective endothelial–mesenchymal transition (EMT) in their outflow tracts [69,70,71].

#### 3.5.2. Inflammatory Response and Atherosclerosis

The predicted dysregulation of downstream targets following the inhibition of *SIRT1* and *ETS1* was also associated with increased inflammation and atherosclerosis, as validated by animal models studies. Myeloid cell-specific *SIRT1*^−/−^ mice cultivated hyperacetylated NF-kB, thus resulting in increased the transcriptional activity of proinflammatory genes [72]. The model prediction that *SIRT1* inhibition would directly activate *VEGFD*, thus inciting the inflammatory response, as previously described by induced mast cell chemotaxis [73]. *ETS1* inhibition was predicted to inhibit *HGF*, thus resulting in predicted inflammation. HGF has been found to suppress the inflammatory response by suitably regulating proinflammatory IL-6 and antiinflammatory IL-10, as previously demonstrated in mouse bone marrow-derived macrophages [74]. The direct inhibition of *CDH5* following *ETS1* inhibition led to the predicted activation of *SHC1*-induced atherosclerosis in this model. Correspondingly, p66^Shc−/−^ mice have presented reduced oxidative stress, vascular apoptosis, and early atherogenesis [75]. The activation of proinflammatory and proatherogenic CRP was also predicted to occur through *ETS1* inhibition via *HGF*, *ITGB1*, and *KLF2*.

#### 3.5.3. Influence of Metformin

The introduction of metformin to the IPA model further explicated its downregulation of miR-199b-5p and demonstrated its cardio-protective role by reversing the inhibition of *SIRT1*, *ETS1*, and *JAG1* and their subsequent downstream effects. Additionally, the direct associations of metformin with *HBA1*, *CRP*, and *IGF1* inhibition were predicted. Metformin was predicted to ameliorate hyperglycemia, inflammation, and atherosclerosis, in addition to restoring angiogenesis and vasculogenesis, in the diabetic state. The downregulation of miR-199b-5p by metformin was predicted to occur via the suppression of the NFATC (via *RELA*) and STAT3 pathways. The increased expression of *STAT3* was found to upregulate miR-199a-1 in murine models [76], and previous publications have reported the relationship between miR-199b and NFATC signaling [30]; however, further research is required to validate these mechanisms. Furthermore, the mere predictive basis of this IPA model necessitates additional in-depth studies.

### 3.6. Clinical Applications for CVD

The relevance of this study concurs with the extensive ongoing research into developing novel CVD therapies. These findings strengthen previously published propositions targeting the downstream target genes implicated in CVD and contribute to the identification of targeted miRNA-based therapies.

#### 3.6.1. miR-199b-5p

The inhibition of antiangiogenic/proatherogenic miR-199b-5p possesses potential as a novel therapy for T1DM and its cardiovascular complications. The antagomir silencing of miR-199b-5p in transgenic mouse models of myocardial infarction resulted in attenuated cardiac dysfunction [77]. Nonetheless, there are limited studies investigating the direct effects of miR-199b-5p inhibition on atherosclerosis.

#### 3.6.2. SIRT1

SIRT1 is required for EC function and vascular angiogenic activity, as the miRNA-mediated epigenetic silencing of *SIRT1* was attributed to endothelial migratory defects and impaired vasculogenesis [78]. Animal models have similarly implicated SIRT1 in the regulation of EC proliferation and senescence [79], thus signifying a protective role of SIRT1 in atherosclerosis.

#### 3.6.3. ETS1

ETS1 induction is fundamental for the regulation of proangiogenic genes [66], which was supported by research reporting that the direct suppression of the ETS1/MMP1 pathway by miR-199a-5p results in reduced angiogenesis [48]. A possible mechanism of ETS1 in attenuating atherosclerosis has also been described via the mediation of 17b-oestradiol [80].

#### 3.6.4. JAG1

Studies by High et al. [70,71] have strongly associated JAG1 with vascular remodeling and VSMC development in both murine and human cell lines. Defective EMT was observed in *JAG1*^−/−^ mice and has been linked with cardiac fibrosis [81], thus further signifying the importance of *JAG1* regulation in cardiovascular health.

### 3.7. Contribution/Causation

Based on the correlation analysis, this research has identified the possible contributions or causations generated from miR-199b-5p target gene binding sites, as listed in Table 1. The utilization of TargetScanHuman, release 8.0, enabled the further exploration of the relevant target genes for miR-199b-5p, and applicable tools within IPA established the most appropriate functional pathways associated with these genes. miR-199b-5p has binding sites in three target genes of interest. A causal effect was demonstrated with respect to SIRT1, which regulates the inflammatory response and enhances angiogenesis. Causal effects were also observed with respect to ETS1 and JAG1, both of which contribute to angiogenesis and vasculogenesis. This study has identified key potential therapeutic targets for metformin therapy in subclinical CVD, thus providing future studies with the fundamental blocks to ultimately prove the causal role of miR-199b-5p on its IPA-predicted target genes.

## 4. Materials and Methods

### 4.1. Study Design

The role of miR-199b-5p in subclinical CVD was explored in a cross-sectional study undertaken using the available sample from a case control open label intervention study, which was previously published by Ahmed et al. [1]. The present study recruited 29 individuals with T1DM as the TG and 20 age- and sex-matched HCs. TG inclusion criteria were satisfactory glycemic control (HbA1c < 8.5% [69 mmol/mmol]) and absence of macro/microvascular disease or diabetic complications (stage 3b renal impairment, i.e., eGFR < 45 mL/min/1.73 m^2^: active proliferative retinopathy). The exclusion criteria included suspected hypoglycemia unawareness. Following a run-in phase of six weeks, the TG was given metformin for eight weeks with a 500 mg dose titrated up to a maximum of 1 g twice daily over 2–3 weeks or to the maximum tolerated dose.

Written informed consent was obtained from all subjects prior to their participation in the study. The study was conducted in accordance with the Helsinki Declaration and received ethical approval from the NHS Health Research Authority, NRES Committee Northeast-Sunderland, UK (REC Ref. 12/NE/0044). Individuals with T1DM were recruited either from Queen Elizabeth Hospital, Gateshead, or Royal Victoria Infirmary, Newcastle, and HCs comprised staff from the aforementioned hospitals and students from Newcastle University, UK.

### 4.2. Clinical and Laboratory Methods

For all study participants, routine laboratory investigations and clinical examinations were performed at baseline and on completion of the study. Unchanged glycemic control was assessed by HbA1c at four time points over 14 weeks and ensured in metformin patients by continuous glucose monitoring for 48 h minimum. EasyGV^®^ 8.8.2 R2 software calculated glucose variability index, as per Hill et al. [82].

Fasted peripheral EDTA blood samples were collected at baseline for HCs and TG, as well as at the end of the study for TG. Processing occurred within four hours of collection, and isolated plasma was used for metabolic and biomarker tests.

### 4.3. Meso Scale Discovery (MSD) Assay for Cytokine Analysis

Cytokines in plasma samples from individuals with T1DM and HCs were diluted in accordance with the manufacturer’s protocol and assayed using K15050D V-PLEX Cytokine Panel 1 Human Kit, K15049 V-PLEX Pro-inflammatory Panel 1 Human Kit, K15190D V-PLEX Angiogenesis Panel 1 Human Kit, K15135C V-PLEX Human Vascular Injury 1 Kit, K15198D V-PLEX Vascular Injury Panel 2 Human Kit, and K151JFC Human TIMP-1 Kit (MSD, Rockville, MD, USA). The plates were read using MSD Sector Imager 2400, and data were analyzed using MSD Workbench 2.0 software.

### 4.4. IGF-1 and IGFBP-3 Enzyme-Linked Immunosorbent Assay

Serum levels of IGF-1 and IGFBP-3 from T1DM and HCs were measured using the Human IGF-I/IGF-1 Quantikine^®^ ELISA Kit (R & D Systems, Minneapolis, MN, USA) and Human IGFBP-3 Quantikine^®^ ELISA Kit (R & D Systems), respectively, according to the manufacturer’s protocol and as previously described [38].

### 4.5. Flow Cytometric Evaluation of Circulating Endothelial Progenitor Cells

cEPCs (CD45^dim^CD34^+^VEGFR-2^+^ cells) and cECs (CD45^dim^CD133^−^CD34^+^CD144^+^ cells) were evaluated as previously described by Ahmed et al. [1]. The sample was analyzed using flow cytometry on BD FACS Canto™ II system, and the results were evaluated using BD FACSDiva™ software (BD Biosciences, San Jose, CA, USA).

### 4.6. Culture and Quantification of CFU-Hill’s Colonies

CFU-Hill’s colonies (described by Hill et al. [2]) were cultured and quantified according to Ahmed et al. [1]. CFU-Hill’s colonies were identified as clusters of rounded cells emanating multiple spindle-shaped cells.

### 4.7. Real-Time Quantitative Polymerase Chain Reaction (RTqPCR) for miRNA Expression

Total RNA was isolated using miRNeasy Micro Kit (QIAGEN, Hilden, Germany) according to the manufacturer’s instructions. Analysis of miRNA expression in CFU-Hill’s was performed using the miRCURY LNA miRNA PCR Human panel I + II (Catalog number: 339322, QIAGEN), as conducted by QIAGEN Genomic Services (QIAGEN). A “no template” sample was included in the RT step as a negative control. For quality control, RNA isolation controls UniSp2,4–5 were added to the purification, cDNA synthesis control UniSp6 was added in the reverse transcription reaction, and DNA spike-in UniSp3 was present on all panels.

Approximately, 10 μL RNA was reverse transcribed in 50 μL reaction using the miRCURY LNA RT Kit protocol (QIAGEN); then, the resulting cDNA was diluted 100 fold and assayed in 10 μL PCR reactions according to the miRCURY LNA miRNA PCR protocol. miRNAs were assayed by qPCR on the miRNA Ready-to-Use PCR, as well as the Human panel I + II, using miRCURY LNA SYBR Green master mix. The LightCycler^®^ 480 Real-Time PCR System (Roche, Basel, Switzerland) was utilized to perform amplification, and amplification curves were analyzed using the Roche LC software 4. Cq values were calculated as the second derivative, and all data were normalized using the global mean method (yielding ΔCq), which was performed based on the average of the assays detected in all samples (169 assays in present study). Fold-change analysis of relative miRNA expression was performed using 2^−ΔΔCq^ calculation, with ΔΔCq obtained from (ΔCq × T1DM) − (ΔCq × HCs).

### 4.8. Ingenuity Pathway Analysis (IPA) of miR-199b-5p

The target genes, cellular functions and diseases, and canonical and pathological pathways regulated by miR-199b-5p were predicted, and interactive models of experimental systems were built by using IPA software 9.0 (QIAGEN Ingenuity^®^, Redwood City, CA, USA). IPA uses content from miRBase, TargetScan, and the Ingenuity Knowledge Base to model relationships and compose molecular networks between miRNAs and their targets, as well as other molecules, diseases, and functions. Interaction sites between the transcripts and miR-199b-5p were identified using TargetScanHuman, release 8.0 (www.targetscan.org/ accessed on 28 February 2024), and Diana-TarBase v.8 (https://dianalab.e-ce.uth.gr/html/diana/web/index.php?r=tarbasev8/index/ accessed on 28 February 2024) databases.

### 4.9. Statistical Analysis

Data presented as mean ± standard deviation (SD) for all analysis. Shapiro–Wilk test was performed prior to analysis to assess the normality of the data. To measure the differences of miR-199b-5p expression between study groups, either one-way analysis of variance (ANOVA) or the Kruskal–Wallace test was performed. Adjustments for multiple comparisons were made using Tukey’s or Dunn’s correction. Comparisons of phenotypic data between two study groups were analyzed using either the unpaired t-test or Mann–Whitney test. Correlations between miR-199b-5p expression and other markers were examined using either the Spearman or Pearson test and linear regression analysis. ROC curve analysis was carried out to assess the sensitivity and specificity of miR-199b-5p and other parameters as biomarkers for subclinical CVD, as well as establishing the cutoff value for miR-199b-5p upregulation. Statistical analyses were performed, and graphs were constructed using GraphPad Prism 9.0 (GraphPad software, San Diego, CA, USA). A *p* value less than 0.05 was considered statistically significant.

## 5. Limitations

A limitation in this study are the technical restraints encountered in attaining sufficient RNA from CFU-Hill’s colonies in view of analyzing the miRNA in parallel with the RNA of individual subjects. Consequently, it was not possible to study the effect of metformin on cells’ receptors and the mechanism by which metformin downregulates miR-199b-5p. Several samples produced skewed/non-normal data and displayed no significant correlation with clinical characteristics despite demonstrating tendencies toward a trend. This could equally be the result of small sample sizes. Nonetheless, this study is the first to assess miR-199b-5p expression in CFU-Hill’s colonies and thus should be regarded as a pilot study from which the feasibility of miR-199b-5p-based CVD therapy can be researched further.

## 6. Conclusions

Our findings substantiate the potential of exploiting miR-199b-5p as a biomarker of CVD and its downstream genes as therapeutic targets for novel CVD research. They also support the therapeutic targets and the mechanism of cardio-protective benefit regarding metformin therapy; however, as this is predictive, further research is required for validation in clinical studies.

## Figures and Tables

**Table 1:** The predicted consequential pairing of miR-199b-5p and the target region in the transcript.

Target Gene	Representative Transcript	Gene Name	Transcript Position	Predicted Consequential Pairing of Target Region Transcript (Top) and miRNA (Bottom)	Site Type
SIRT1	ENST00000212015.6	Sirtuin 1	507–513 3′UTR	5′CACAAUUAUUU-UUAA**ACACUGG**C 3′CUUGUCCAUCA-GACU**UGUGACC**C	7mer-m8
ETS1	ENST00000531611.1	ETS Proto-Oncogene 1, Transcription Factor	2886–2893 3′UTR	5′UGGUGGGUGGU-UUAU**ACACUGG**A 3′CUUGUCUAUCA-GAUU**UGUGACC**C	8mer
JAG1	ENST00000254958.5	Jagged Canonical Notch Ligand 1	135–141 3′UTR	5′UUGACAAGCUG-GCUU**ACACUGG**C 3′CUUGUCUAUCA-GAUU**UGUGACC**C	7mer-m8

TargetScanHuman, release 8.0 (www.targetscan.org [accessed on 28 February 2024]) database was used to predict the interaction site between the target transcripts and miR-199b-5p. Nucleotides in **bold** represent the predicted consequential pairing of target region transcript and miRNA.

## Data Availability

The original contributions presented in the study are included in the article; further inquiries can be directed to the corresponding author.
